# Successful treatment of Heyan Kuntai capsule combined with hormonal therapy in an adolescent with diminished ovarian reserve: a case report

**DOI:** 10.1186/s13048-025-01716-0

**Published:** 2025-07-03

**Authors:** Xue Ao, Ling Xiong, Jialu Zhu, Shiqiao Tan

**Affiliations:** 1 Departments of Obstetrics and Gynecology, West China Second University Hospital, Sichuan University, Chengdu, People’s Republic of China; 2 Key Laboratory of Birth Defects and Related Diseases of Women and Children, Sichuan University, Ministry of Education, Chengdu, People’s Republic of China; 3 Shanghai Haitian Pharmaceutical Technology Development Co., Ltd, Shanghai, People’s Republic of China

**Keywords:** Heyan Kuntai capsule, Diminished ovarian reserve, Adolescent, Hormone therapy, Case report

## Abstract

**Background:**

Diminished ovarian reserve (DOR), a common female disease, results in reduced fertility and menstrual disorders. Patients without timely treatment often suffer from infertility, ovarian insufficiency, premature ovarian failure and early onset or worsened perimenopausal symptoms caused by estrogen fluctuation or deficiency. However, there is a lack of effective methods to improve ovarian reserve function.

**Case presentation:**

Here, we report a case of a 13-year-old female with 2-year menstrual disorder. The anti-mullerian hormone (AMH) levels were test twice, with interval of 2 months, yielding results of 0.41 ng/mL and 0.50 ng/mL, respectively. Colour Doppler ultrasound examination showed that there were 4–6 antral follicles bilaterally. The patient was diagnosed with DOR. She was treated with Heyan Kuntai capsule (HYKT) combined with hormonal therapy (HT). Post treament, her AMH level, follicle stimulating hormone level and AFC normalized. Discontinuation of HYKT and continuation of HT alone for 5 months proved ineffective. Consequently, HYKT in combination with HT was continued, leading to normal ovarian reserve function restoration. No adverse reactions were observed during the 4-year follow-up.

**Conclusions:**

This case highlights the importance of including DOR as a diagnostic consideration in adolescents with menstrual disorders during clinical evaluation. AND combining HYKT with HT might contribute to potential improvements in ovarian function in DOR patients.

## Background

Diminished ovarian reserve (DOR) is characterized by ovarian insufficiency due to a reduction in the quantity and/or quality of oocytes resulting in reduced fertility, and accompanied by decreased anti-mullerian hormone (AMH) level, decreased antral follicle count (AFC), and elevated follicle stimulating hormone (FSH) level [1]. The diagnosis of DOR depends on a comprehensive assessment of the above indicators, and is usually indicated by AMH < 1.1ng/ml, bilateral ovarian AFC < 5–7, and basic FSH ≥ 10 IU/L for two consecutive menstrual cycles, excluding drug, physiological, lifestyle and other factors [2]. DOR prevalence is over 50% in women over 40, and about 10% of women may experience an early reduction in ovarian reserve regardless of age for various reasons [3]. At present, only a few literatures have reported adolescent DOR caused by chemotherapy [4, 5], chromosomal abnormality [6] or ovarian cysts [7]. The pathogenesis of DOR is still unclear, and there is no unified treatment strategy. Individualized treatment is often used in clinical practice, which mainly includes hormone therapy (HT), ovulation induction and traditional Chinese medicine, etc. HT and ovulation induction can significantly improve symptoms, while long-term use may carry risks such as thrombosis and gynaecological cancers [8]. In Chinese clinical practice, Traditional Chinese medicine (TCM) is recommended to improve the ovarian function of patients with DOR [2].

Heyan Kuntai capsule (HYKT) was approved by the National Medical Products Administration for the treatment of diseases or symptoms associated with diminished ovarian function in 2000. It is a traditional Chinese medicinal formula, originates from the Huang Lian E Jiao decoction recorded in the Shang Han Lun. HYKT is formulated with Rehmanniae Radix Praeparata (*Rehmannia glutinosa (Gaertn.)DC.*), Rhizoma Coptidis (*Coptis chinensis Coptis chinensis Franch.*), Paeoniae Radix Alba (*Paeonia lactiflora Pall.*), Colla Corii Asini (*Asini Corii Colla*), Scutellariae Radix (*Scutellaria baicalensis Georgi.*), and Poria (*Poria cocos(Schw.)Wolf.*). According to traditional Chinese medicine, the pathological mechanism of DOR is “spleen and kidney deficiency, insufficient Tian Gui, and deficiency in Chong and Ren meridians.” The formula features Rehmanniae Radix Praeparata (containing iridoid glycosides, polysaccharides, and amino acids) as the monarch herb for nourishing essence, replenishing kidney yin, and enriching blood; the minister herbs include Colla Corii Asini (rich in collagen and its hydrolysates) and Paeoniae Radix Alba (with monoterpene glycosides like paeoniflorin and albiflorin) to synergistically enhance blood and yin supplementation, along with Rhizoma Coptidis (alkaloids such as berberine and palmatine) to clear deficiency-fire and calm irritability, collectively achieving yin-nourishing, fire-reducing, and heart-kidney coordination; Assistant herbs Poria (with pachyman and triterpenes) and Scutellariae Radix (flavonoids like baicalin and baicalein) further support spleen-strengthening, mind-tranquilizing, and heat-clearing effects, reinforcing the formula’s action on fire reduction and heart-kidney interaction [9]. The combination of these six herbs has the effect of nourishing *Yin*, nourish blood, clearing heat, tranquilizing the mind, and eliminating vexation [9].

Here, we report a rare case of adolescent DOR patient. The ovarian reserve function was improved after treatment with Heyan Kuntai capsule (HYKT) combined with HT, and during the 4-year follow-up period, no adverse reactions were found. This case shows the possibility of using HYKT combined with HT as an option for DOR treatment.

## Case presentation

The female patient, 13 years and 7 months old, was admitted to the hospital on November 08, 2019, complaining of menstrual disorder for 2 years. Her first menarche occurred at the age of 11. She had no known allergies and sexuality. In July 2019, she was hospitalized due to abnormal uterine massive bleeding. While the AMH level was 0.41 ng/mL, and the routine chromosome examination and G-banding karyotype analysis showed 46, XX. The patient received drospirenone and ethinylestradiol tablets (II) for menstrual regulation. On October 21, 2019, the AMH level was 0.50 ng/mL, the estradiol (E2) level was 23 pg/mL, and the FSH level was 2 IU/L, indicating DOR. The patient was given HYKT orally, 2.0 g three times daily, along with oral dydrogestrone, 10 mg twice daily for 10 days. On February 14, 2020, the results of the color Doppler ultrasound examination of the patient showed no obvious abnormality in the uterus and accessories, and 4 ~ 6 follicles were seen inside. There was no obvious abnormality in breast color ultrasound. Her AMH levels were 0.8 ng/mL, E2 levels were 200.2 pg/mL, and FSH levels were 7.1 IU/L. Combined with AFC, the patient was confirmed the diagnosis of DOR by comprehensive assessment. Then, the patient was given HYKT orally, 2.0 g three times daily, along with oral dydrogestrone, 10 mg twice daily for 2 months.

In the subsequent follow-up, HYKT combined with HT continued to be used, and the ovarian reserve function of the patients were improved compared with those before treatment. The results of the hormone examination on June 26, 2021 showed that an elevated AMH level was 1.17 ng/mL, the E2 level was 57.5 pg/mL, and the FSH level was 7.1 IU/L. Color Doppler ultrasound examination of uterine adnexa showed 9 ~ 11 AFCs. Liver and kidney function and breast color ultrasonography showed no obvious abnormality. The patient discontinued HYKT on July 16, 2021 and was only given drospirenone and ethinylestradiol tablets (II) orally, 1 tablet (containing 0.02 mg ethinylestradiol/3 mg drospirenone) once daily for 5 months.

After 5 months of HT alone (December 10, 2021), the AMH level decreased to 0.74 ng/mL, the E2 level was 19.1 pg/mL, and the FSH level was 8.5 IU/L. Ultrasound of the uterine adnexa showed no definite developing follicle in the right ovary and the left ovary was not clear. In view of the unsatisfactory efficacy, the treatment was changed to HYKT orally, 2.0 g three times daily, along with complex packing estradiol tablets/estradiol and dydrogesterone tablets (2 mg:10 mg*28 tablets) orally, 1 tablet once daily, for 5 months.

On August 18, 2022, the hormone test results showed that the AMH level increased to 3.59 ng/mL, the E2 level was 42.4 pg/mL, and the FSH level was 5.5 IU/L. Color ultrasound of uterine adnexa showed 12 ~ 14 AFCs. A color ultrasound examination of the breast showed a weak echo in the left breast (Breast Imaging Reporting and Data System [BI-RADS] 3). Liver and kidney function showed no obvious abnormality. The patient was given HYKT orally, 2.0 g 3 times daily, along with drospirenone and ethinylestradiol tablets (II) orally, 1 tablet (containing 0.02 mg ethinylestradiol/3 mg drospirenone) once daily for 1 year, and the patient stated that HYKT was taken intermittently during this period.

One year later (August 2023), the AMH level was 0.81 ng/mL, the E2 level was 23.6 pg/mL, and the FSH level was 3.2 IU/L. The right ovary was normal in size, with 5–6 follicles seen, with a maximum diameter of 0.7 cm. The left ovary was normal in size, with 5–6 follicles seen, with a maximum diameter of 0.8 cm. Liver and kidney function and breast color ultrasonography showed no obvious abnormality. The patients was given HYKT orally, 2.0 g three times daily, along with complex packing estradiol tablets/estradiol and dydrogesterone tablets (2 mg:10 mg*28 tablets) orally, 1 tablet once daily, for 6 days.

At the final follow-up in March 2024, the ovarian reserve function had significantly improved, with an AMH level of 3.86 ng/mL, an E2 level of 50.4 pg/mL, and an FSH level of 7.2 IU/L. The right ovary was normal in size with multiple follicles (5–6 per section) with a maximum diameter of 0.7 cm. The left ovary was normal in size with multiple follicles (4–5 per section) with a maximum diameter of 0.5 cm. Liver and kidney function showed no obvious abnormality. Through continuous treatment and follow-up for more than 4 years, the ovarian function of the patient was gradually recovered and finally reached the ideal state. No adverse reactions were found.

**Fig. 1:**
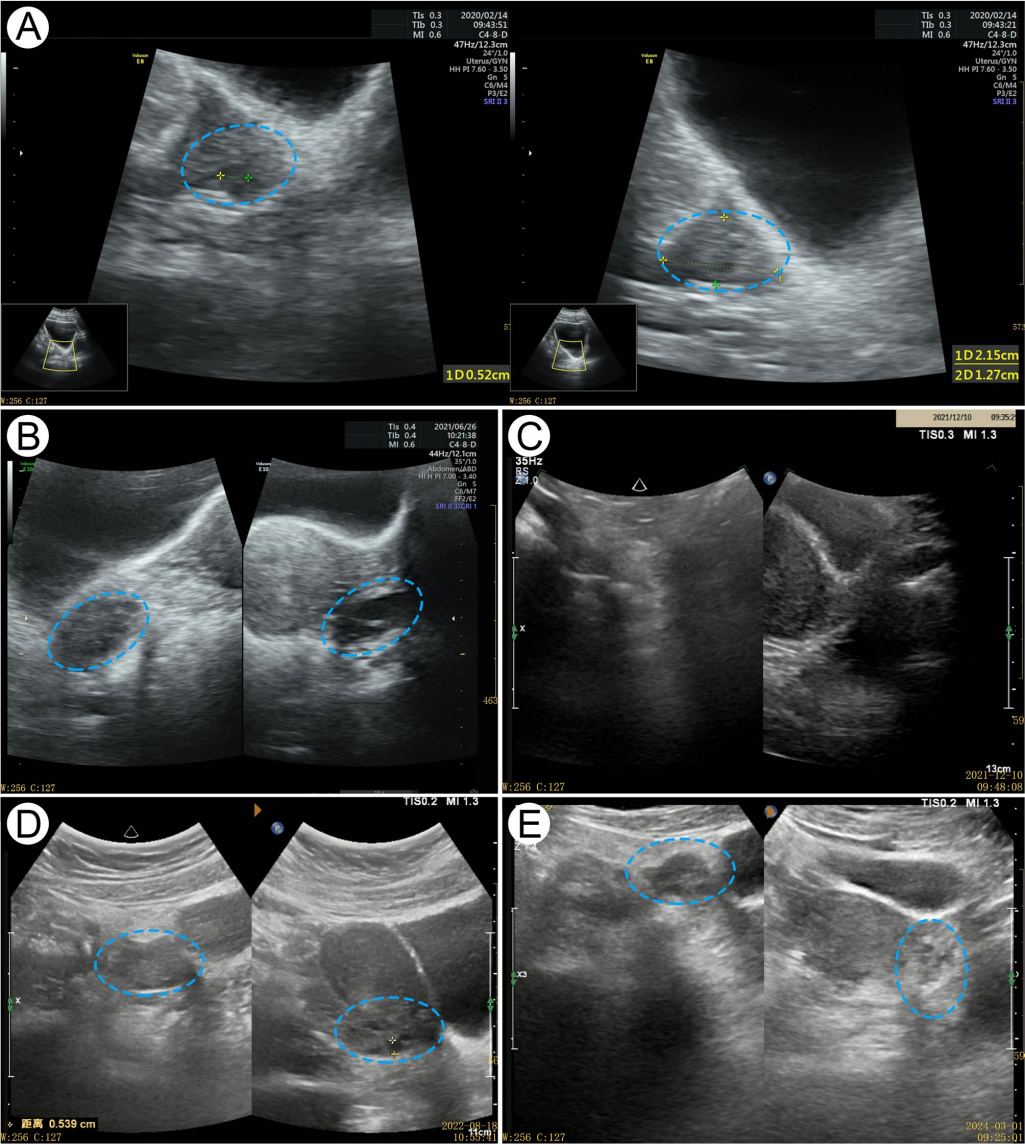
Ultrasound pictures of the patient’s ovarian. The blue frame indicates the changes in the patient’s ovaries: **A** Baseline (2020.02.14): Bilateral ovaries with few follicles (2–3 per side). **B** Before discontinuation of Heyan Kuntai capsule (HYKT) (2021.6.26): The right ovary measured 2.7 × 1.8 × 2.5 cm with 5–6 follicles (max 0.7 cm), and the left ovary measured 2.6 × 1.2 × 2.0 cm with 4–5 follicles (max 0.9 cm). **C** After discontinuation of HYKT (2021.12.10): Ovaries shrank with no visible follicles. **D** After adding HYKT (2022.8.18): Unilateral antral follicles and ovarian volume increased. **E** Final follow-up (2024.3.1): The right ovary (5–6 follicles, max 0.7 cm) and the left ovary (4–5 follicles, max 0.5 cm) were normal in size

The changes of AMH, E2 and FSH levels, the changes of uterine adnexa and breast color ultrasound, and the results of liver and kidney function of patients at each time point are shown in Fig. 1; Table 1.

**Table 1:** Timeline of treatment and laboratory/imaging findings

Date	AMH (ng/mL)	E_2_ (pg/mL)	FSH (IU/L)	Uterine adnexa					Breast		Liver and kidney function	Medications	Course of treatment
				Ovarian size (cm)		Antral follicle count		Ultrasound diagnosis	Ultrasound description	Ultrasound diagnosis			
				Right	Left	Right	Left						
**2019.07.08**	0.41	–	–	–	–	–	–	–	–	–	–	–	–
**2019.10.21**	0.5	23	2	–	–	–	–	–	–	–	–	–	–
**2019.11.08**	–	–	–	–	–	–	–	–	–	–	–	HYKT + dydrogestrone	10 days
**2020.02.14**	0.8	200.2	7.1	1.8 × 1.0 × 1.9	2.2 × 1.3 × 1.7	2 ~ 3	2 ~ 3	No obvious abnormality	The echogenicity of bilateral breast glands was not homogeneous, with no exact localisation, and no obvious abnormal blood flow signals were detected. There was no definite growth of lymph nodes in the axilla bilaterally.	No lesions in both breasts	–	–	–
**2020.05.22**	–	–	–	–	–	–	–	–	–	–	–	HYKT + dydrogestrone	2 months
**2020.06.15**	1.31	55.9	5.6	2.3 × 1.7 × 2.4	2.6 × 1.5 × 1.8	3 ~ 4	4 ~ 5	No obvious abnormality	–	–	–	–	–
**2020.07.03**	–	–	–	–	–	–	–	–	–	–	–	HYKT + dydrogestrone	3 months
**2020.10.29**	1.16	33.9	6.5	2.8 × 1.6 × 2.5	2.7 × 2.0 × 2.2	7 ~ 8	6 ~ 7	No obvious abnormality	The echogenicity of bilateral breast glands was not homogeneous, with no exact localisation, and no obvious abnormal blood flow signals were detected. There was no definite growth of lymph nodes in the axilla bilaterally.	No lesions in both breasts	–	–	–
**2020.11.04**	–	–	–	–	–	–	–	–	–	–	–	HYKT + dydrogestrone	3 months
**2021.01.18**	0.74	86.8	6.9	3.0 × 1.7 × 1.9	3.1 × 1.7 × 1.8	5 ~ 6	6 ~ 7	No obvious abnormality	–	–	–	–	–
**2021.01.29**	–	–	–	–	–	–	–	–	–	–	–	HYKT + dydrogestrone	6 months
**2021.06.26**	1.17	57.5	7.1	2.7 × 1.8 × 2.5	2.6 × 1.2 × 2.0	5 ~ 6	4 ~ 5	No obvious abnormality	The echogenicity of bilateral breast glands was not homogeneous, with no exact localisation, and no obvious abnormal blood flow signals were detected. There was no definite growth of lymph nodes in the axilla bilaterally.	No lesions in both breasts	Negative	–	–
**2021.07.16**	–	–	–	–	–	–	–	–	–	–	–	drospirenone and ethinylestradiol tablets (II)	5 months
**2021.12.10**	0.74	19.1	8.5	2.2 × 1.2 × 1.2	Not clear	No definite follicles		No obvious abnormality	–	–	–	–	–
**2021.12.17**	–	–	–	–	–	–	–	–	–	–	–	HYKT + complex packing estradiol tablets/estradiol and dydrogesterone tablets	5 months
**2022.08.18**	3.59	42.4	5.5	2.8 × 1.7 × 2.8	2.4 × 1.6 × 2.5	7 ~ 8	5 ~ 6	Please consider clinical presentations	The echogenicity of the glandular layer of both breasts was not homogeneous, and a weak echo measuring 1.9 × 1.6 × 1.8 cm was detected in the left breast next to the nipple at 1 o’clock, the border was clear, and no obvious blood flow signal was detected. In the right breast, there was no definite localisation, and no obvious abnormal blood flow signal was detected. There was no definite growth of lymph nodes in the axilla bilaterally.	Left breast weak echo (BI-RADS 3)	Negative	–	–
**2022.08.20**	–	–	–	–	–	–	–	–	–	–	–	HYKT + drospirenone and ethinylestradiol tablets (II)	3 months
**2023.03.03**	1.09	13.1	9.9	Normal	Normal	7 ~ 8	6 ~ 7	Please consider clinical presentations	–	–	–	–	–
**2023.03.10**	–	–	–	–	–	–	–	–	–	–	–	HYKT + drospirenone and ethinylestradiol tablets (II)	3 months
**2023.08.18**	0.81	23.6	3.2	3.0 × 1.6 × 1.8	2.8 × 1.6 × 2.6	5 ~ 6	5 ~ 6	No obvious abnormality	The echogenicity of bilateral breast glands was not homogeneous, with no exact localisation, and no obvious abnormal blood flow signals were detected. There was no definite growth of lymph nodes in the axilla bilaterally.	No lesions in both breasts	Negative	–	–
**2023.08.25**	–	–	–	–	–	–	–	–	–	–	–	HYKT + complex packing estradiol tablets/estradiol and dydrogesterone tablets	6 days
**2024.03.01**	3.86	50.4	7.2	Normal	Normal	5 ~ 6	4 ~ 5	No obvious abnormality	–	–	Negative	–	–

AMH: anti-mullerian hormone; E2: estradiol; FSH: follicle stimulating hormone; HYKT: Heyan Kuntai capsule; BI-RADS: Breast Imaging Reporting and Data System

## Discussion and conclusions

DOR severely affects women’s fertility. The causes are complex, and may be related to a number of factors, including age, genetics, medical factors, autoimmunity, infections, and environmental and psychosocial factors [2]. According to the different age of onset, DOR can be divided into two categories: physiological DOR related to advanced age and pathological DOR incompatible with age [10]. AMH is secreted specifically by granulosa cells of preantral and small antral follicles. Unlike FSH or E2 which show cyclical fluctuations and are tightly regulated by HPO axis feedback mechanisms, AMH exhibits relatively stable levels across menstrual cycles and serves as a more direct and reliable marker of ovarian reserve. AMH concentration begins to rise in infancy, peaks at approximately 25 years, then gradually declines in parallel with the progressive reduction in ovarian reserve, and finally decreases to undetectable levels at menopause [11]. The results of a large-scale cross-sectional study involving 6,763 Chinese women aged 0–51 years and older found that, during childhood and adolescence, AMH level increased, reaching a peak at 18 years, and the reference range of AMH levels in adolescent females aged 11 to 18 years was 1.52–9.41 mg/L [12]. However, the AMH level in the patient reported here was abnormally low, suggesting DOR. There are fewer reports of DOR in adolescence. Chemotherapy-related adolescent DOR was reported by Pruett M et al. This study analysed AMH levels in 37 adolescent cancer survivors treated with heavy metal chemotherapy and showed that more than 1 year after treatment, 8 (21.6%) cancer survivors, aged 13.9–21.3 years, all treated with ≥ 400 mg/m^2^ cisplatin, had low AMH levels (< 0.25–1.28 ng/mL), suggesting DOR [5]. Another study included 102 adolescent and young adult females aged 13.5–41.7 years, and 8–24 months after chemotherapy, 79.4% of patients had AMH levels < 1 ng/mL, suggesting DOR [4]. Genetic factors are also important in pathological DOR, often accompanied by a familial predisposition, especially in families with sex chromosome abnormalities, such as a family history of fragile X syndrome. In addition, gene polymorphism (such as *GDF9* and *FSHR*), gene mutation (*FMR1*), epigenetic factors and chromosome translocation may be involved in the development of pathological DOR [2]. Parissone F et al. reported a case of Xq duplication with short stature and DOR in an 18-year-old girl, who received effective recombinant growth HT and fertility preservation strategies [6]. The chromosomal findings of the adolescent DOR case reported in this paper showed normal results without a history of specific medication. The pathogenesis is still unclear and needs to be further investigated. In conclusion, the etiology of DOR is complex and needs to be analysed comprehensively by combining clinical manifestations, laboratory tests, family history and other information. An in-depth exploration of the pathogenesis of the relatively rare case of adolescent DOR will help to improve the understanding, diagnosis and treatment of this disease.

At present, HT is commonly used for DOR in clinical practice. The mechanism of HT involves exogenous estrogen acting through negative feedback regulation on the HPO axis to reduce FSH levels, thereby restoring follicular sensitivity to endogenous FSH to promote follicular development and ovulation while decreasing excessive follicle recruitment to preserve ovarian reserve and delay ovarian aging [13]. However, HT alone can not achieve satisfactory therapeutic results [2, 14], and many studies have attempted to improve ovarian function using traditional Chinese medicine. For example, a Bayesian network meta-analysis demonstrated that Chinese patent medicines (like Zuogui pill, Xuefu Zhuyu capsule, Ziheche capsule and HYKT) combined with HT were superior to HT alone in improving clinical outcomes in premature ovarian failure, including reducing FSH and LH, enhancing estradiol levels, and minimizing adverse reactions [15]. A meta-analysis of 12 randomized controlled trials involving 905 patients with DOR showed that HYKT combined with HT was superior to HT alone in improving AFC (MD = 1.34, 95%CI [0.96, 1.72]), AMH (MD = 1.09 ng/mL, 95%CI [0.80, 1.38]) and FSH levels (MD = − 6.90 mIU/mL, 95%CI [-11.91, -1.89], I^2^ = 97%) in patients with DOR [14].

The main component of HYKT, Rehmanniae Radix Praeparata (*Rehmannia glutinosa (Gaertn.)DC.*), exhibits estrogenic-like activity, capable of increasing hormone levels [16]. HYKT can improve ovarian function by regulating Smad2/3/7 expression [17], increasing GDF-9 and EGR-1 levels [18], and modulating the Bcl-2/Bax balance [19], thereby promoting follicular growth and inhibiting apoptosis. In cyclophosphamide-induced DOR rats, HYKT restored ovarian histology, improved blood supply, reduced follicular atresia, increased mature follicles, lowered FSH/LH, and elevated E2/AMH levels [18]. A multicenter randomized controlled trial involving 108 infertile women with DOR demonstrated that after 3 months of treatment, the HYKT group showed significantly greater improvements than the DHEA group in AMH (+ 1.27 ± 0.26 vs. + 0.6 ± 0.11 ng/mL, *P* < 0.05) and AFC (+ 2.56 ± 0.85 vs. + 1 ± 0.45, *P* < 0.05) [20]. A retrospective study of 130 infertile women with DOR found that after 3 months of treatment, the increase of AMH and AFC and the decrease of FSH in the HYKT group were significantly higher than those in the Femoston group (*P* < 0.05) [21]. In this case report, the patient presented with menstrual disorders during puberty and was diagnosed with DOR. After HYKT combined with HT, the ovarian reserve function were improved. When HYKT was discontinued, the effect of HT alone for 5 months was not satisfactory. The patient’s AMH level, follicle stimulating hormone level and antral follicle count returned to normal after the combination of HYKT again. During the long-term use of HYKT (more than 4 years), the patient’s liver and kidney function and breast examination were not abnormal. This case shows that HYKT combined with HT might be a pormising approach for treating DOR with no significant side effects. However, the findings should be interpreted with caution due to the inherent limitations of a single case report, including potential confounding factors, lack of control group, and possible individual variability in treatment response.

In conclusion, this article reports a rare case of adolescent DOR successfully treated by HYKT combined with HT, which provides a valuable reference for clinical practice. This case suggests that patients with menstrual disorders and low AMH levels during adolescence should be alert to the possibility of DOR. HYKT combined with HT may be an possible therapeutic option to improve the ovarian function of patients, and the long-term follow-up results have showed its safety, which should be further explored in clinical practice. In addition, adolescent menstrual disorders and other symptoms should be highly regarded, and timely targeted treatment should be taken to maximize the maintenance of patients’ reproductive health.

## Data Availability

The datasets used and/or analysed during the current study are available from the corresponding author on reasonable request.

## Abbreviations

DOR: Diminished ovarian reserve
AMH: Anti-mullerian hormone
HYKT: Heyan Kuntai capsule
HT: Hormonal therapy
AFC: Antral follicle count
FSH: Follicle stimulating hormone
E2: Estradiol
BI-RADS: Breast Imaging Reporting and Data System
